# The patient journey project in Italian mental health services: results from a co-designed survey on clinical interventions and current barriers to improve the care of people living with schizophrenia

**DOI:** 10.3389/fpsyt.2024.1382326

**Published:** 2024-03-28

**Authors:** Antonio Vita, Stefano Barlati, Matteo Porcellana, Elisa Sala, Jacopo Lisoni, Luisa Brogonzoli, Mauro Emilio Percudani, Rosaria Iardino

**Affiliations:** ^1^Department of Mental Health and Addiction Services, ASST Spedali Civili of Brescia, Brescia, Italy; ^2^Department of Clinical and Experimental Sciences, University of Brescia, Brescia, Italy; ^3^Department of Mental Health and Addiction Services, Niguarda Hospital, Milan, Italy; ^4^Department of Political and Social Sciences, University of Pavia, Pavia, Italy; ^5^Research Department, Fondazione The Bridge, Milan, Italy; ^6^Fondazione The Bridge, Milan, Italy

**Keywords:** early detection, mental health services, patient journey, peer support, prevention, recovery, schizophrenia, stakeholder engagement

## Abstract

**Introduction:**

The Patient Journey Project aimed to analyze the scenario among Italian Mental Health Services (MHS) to understand the clinical interventions that are properly implemented and the ones deserving further implementation to design an effective treatment plan for patients living with schizophrenia (PLWS).

**Methods:**

The 60-items survey was co-designed with all the stakeholders (clinicians, expert patients and caregivers) involved in the Patient Journey and focused on three phases of schizophrenia course: early detection and management, acute phase management, long-term management/continuity of care. Respondents were Heads of the Mental Health Departments and Addiction Services (MHDAS) or facilities directors throughout Italian MHS. For each statement, respondents expressed the consensus on the importance and the degree of implementation in clinical practice.

**Results:**

Considering the importance of the statement, strong consensus was reached for most of the statements. Good levels of implementation were found on 2/17 statements of early detection and management, on 3/16 statements for acute phase management and on 1/27 statements of long-term management/continuity of care. Poor levels of implementation were found on 1/17 statements of early detection and management, none of acute phase management, and 4/27 statements for long-term management/continuity of care. Moderate levels of implementation were found on 14/17 statements for early detection and management, on 13/16 statements of acute phase management, and on 22/27 statements of long-term management/continuity of care. Thus, among Italian MHDAS, most interventions for PLWS were moderately implemented in clinical practice.

**Discussion:**

Italian MHS have to provide new strategies and structural actions to overcome these current limitations and barriers to effectively improve the journey of PLWS. The areas that deserve most implementation include interventions during the early stage (especially the continuity of care between Child and Adolescent Mental Health Services and Adult Mental Health Services), the evidence-based psychosocial interventions during the chronic stages of the disorder, and the continuity of care after acute hospitalization.

## 1 Introduction

### 1.1 Clinical manifestation and economic burdens of schizophrenia

Despite low prevalence rates, schizophrenia is considered among the most severe mental disorders, ranking among the leading causes of disability worldwide (1, 2). Clinically, patients living with schizophrenia (PLWS) experience psychotic, negative, disorganizative symptoms and cognitive impairments, the latter representing the most detrimental factors associated to functional decline (1, 3). Being characterized by a debilitating, multi-episodic and chronic progression in up to 60% of cases (4), schizophrenia is associated with massive social and economic costs for patients, caregivers, society and Mental Health Services (MHS). Indeed, in Italy in 2022, more than a third of psychiatric outpatient services were dedicated to the care of patients with schizophrenia (5). Moreover, if in Europe in 2010 the total cost of psychotic disorders (including schizophrenia) was estimated at 93.9 billion Euros (6), in Italy the economic burden for schizophrenia was estimated at around €2.7 billion (7), with 50.5% (almost €1.39 billion) due to indirect costs and 49.5% to direct costs (almost €1.37 billion). The latter corresponded, respectively, to drugs therapies (accounting for 10% of direct costs) and to hospitalizations (accounting for 81% of direct costs, including residential and semi-residential facilities) (7). Indeed, in Italy, 13.800 patients with schizophrenia were hospitalized from 2009 to 2016, with an average of 2.98 hospitalizations per patient (8). Considering indirect costs, the loss of productivity is an essential topic, involving both personal and caregiver’s functioning: indeed, families members and caregivers lost, on average, 44.1 working days yearly in activities linked to the disorder (7), and this is responsible of severe family burdens and reduced quality of life for relatives and caregivers (9). Moreover, it has been found that, in Italy approximately 15.000 PLWS received some social security benefits yearly from 2009 to 2015, with an average annual expenditure of €160.1 million (8). Indeed, throughout the course of illness, negative symptoms, impairments of adaptive life skills and of cognitive performance are a leading source of disability and altered real-life functioning (3, 10, 11). Furthermore, PLWS are characterized by reduced life expectancy with a weighted average of 14.5 years of life lost (12) and increased mortality rates that are more than double than the general population (13, 14), due to suicides (15) and, mostly, to cardiovascular diseases (16). Moreover, incidence rates of a wide range of somatic disorders (including diabetes mellitus, metabolic syndrome, respiratory, autoimmune disease, infections, cancers) are significantly higher among PLWS than in the general population (17, 18). Consequently, physical health is a major concern in the care of individuals with schizophrenia. Indeed, several external (i.e., accessibility to health care and medication) and internal factors (i.e., self-esteem, negative and cognitive symptoms, alimentation, and substance misuse) are responsible for the scarce physical health of affected individuals (19). However, on one hand, if poor lifestyle habits (e.g., smoking, sub-optimal treatment of somatic disorders) and medications account for much of the increased mortality risk due to somatic diseases (20), on the other hand current evidence from genetic studies suggested that a common shared genetic risk for cardiovascular risk factors and psychotic disorders could explain the increased risk for cardiovascular disorders (20, 21). Among external factors, the role of medications, primary antipsychotic compounds (AP) (first generation antipsychotics, FGA; and second generation antipsychotics, SGA), is controversial: on the one hand, if it is well known that AP are linked to metabolic side effects (including dyslipidemia, hyperglycemia, obesity and overweight, diabetes mellitus, thyroid disorders, hyponatremia) (22), on the other hand highest cumulative mortality rates were observed among those patients with no AP exposure whereas taking AP medication, at adequate dosages, is associated with lower mortality due to somatic comorbidities (23, 24). Moreover, possible different AP administration routes (oral versus long-acting injection, LAI) are other factors that could influence mortality rates in schizophrenia, with second generation long-acting injection (SG-LAI) associated with the lowest cumulative mortality rate and an approximately a 30% lower risk of death compared with oral agents (25).

### 1.2 Treatment challenges for PLWS

An important issue in the care of PLWS is the role of pharmacological interventions, namely AP drugs. Indeed, these agents are relatively effective in improving positive symptoms, such as auditory hallucinations and delusions, but not markedly effective on negative symptoms and cognitive impairments (26). Marcellusi and colleagues found that of the 212.739 individuals diagnosed with schizophrenia in 2014 in Italy, the majority (~86%) were treated with AP pharmacotherapy (7), suggesting that most of patients required long-term, or even lifetime, medications to control their symptoms (1, 26). The authors also found that APs were usually combined with other CNS drugs (polypharmacotherapy) in more than half of the cases (7), confirming that add-on therapies were frequently prescribed in PLWS (27, 28). However, oral AP polypharmacy has been extensively associated to non-adherence phenomenon, to reduced tolerability and increased adverse effects and higher costs for MHS (29, 30). Coherently, in the U.S. in 2005, it was found that the national rehospitalization costs related to antipsychotic non-adherence were, on average, $1479 million (31). Indeed, as relapses can worsen the course and outcomes of the disorder by reducing treatment response and producing severe personal and societal repercussions (11), relapse prevention is essential for the management of schizophrenia for which MHS have to offer plans to reduce those factors contributing to relapse, including drug discontinuation (11). Thus, an effective option is to use LAI that, compared to oral AP, seems to improve the compliance and reduce the risk of relapse and of new hospitalizations (32, 33). Moreover, the involvement of patients in shared decision-making on pharmacotherapy is essential to improve the subjective quality of care (11, 34, 35).

### 1.3 The value of patient journey in a recovery-oriented perspective

Nevertheless, caring for PLWS does not solely end in treating these patients pharmacologically to control the symptoms of the disorder and it cannot be reduced to economic and financial aspects only. Indeed, if AP remain an essential starting point to achieve and maintain symptomatic remission, an effective management also requires that pharmacotherapy is embedded and integrated within a framework of multidisciplinary psychosocial interventions (including cognitive remediation for cognitive impairments, psychological treatments for resistant positive symptoms, family and social support, psychoeducation for patients and families, social skill training, employment services to improve personal work abilities, money management counseling) that have to be delivered in the community-care setting to improve quality of life, satisfaction, well-being and to achieve recovery (1, 4, 11, 26). Among this framework, the network of care should also involve a wide range of professionals and agencies (including, in- and out-patient services, community care centers, self-help groups, family organizations, psychiatrists, psychologists, nurses, social workers, case managers, and general practitioners, GPs) and, obviously, the patient (11). Moreover, it is essential that a congruent care pathway will be built according to a lifespan perspective and, most of all, according to the different stages of the disorder: indeed, three crucial stages have been identified in the course of schizophrenia, that are the prodromal and early phases, the acute states of decompensation and the long-term phases (4, 35). Among these phases, especially in the long-term management of chronicity, the above-cited psychosocial interventions seem to play an essential role improving overall psychosocial functioning (11). Thus, according to the patient’s needs, the value of the treatment has to comprise a combination of symptom reduction, improved quality of life, better social functioning and subjective well-being, and optimal physical health (11), and this is possible if MHS will shared their actions in combination with social systems and with all the stakeholders (including caregivers, self-help groups, family organizations, expert patients and GPs) involved in the everyday life of PLWS (11, 35). However, the ideal path care for PLWS have to address several structural and clinical unmet needs that include the early recognition/intervention plans (to reduce the duration of untreated psychosis), the treatment of resistant symptoms (including negative, depressive and cognitive symptoms), the personalization of pharmacological treatments to maintain remission and reduce non-adherence (weighing the relation between effectiveness and side effects), the need to deal with somatic comorbidities and comorbid substance abuse, and finally, the suboptimal integration of pharmacological and psychosocial interventions and the poor collaboration among health and social care professionals (4, 11). All of these problems concerning the ideal path care of PLWS have already been highlighted in a recent Italian Delphi study showing that, despite a strong consensus on these main components of schizophrenia path care was achieved by the experts, a strong gap exists in the everyday clinical practice on the effective implementation of these themes (4). Detailly, important concerns were found on the dissemination of early prevention/intervention plans, on the lack of structured symptomatologic assessment to guide the personalization of care and of pharmacological treatments (including, switching or augmenting APs, side effects assessment), on the lack of definitive plans to improve treatment adherence, and to monitor cardiovascular/metabolic risk and on the management of somatic comorbidities and physical health (4). Considering these concerns and according to Galderisi et al., 2019 (4), we unfortunately have to observe that the road to organizing an optimal treatment path for PLWS is still long and twisted by several critical barriers that must necessarily be overcome if MHS want to design a definitive treatment plan (11). Indeed, despite recovery is achievable, only 13.5% of affected individuals (1/7 patients) met the defined criteria for a full recovery (36). Recovery is an essential conceptualization that allowed psychiatrists to change the paradigm in treating schizophrenia, moving from a symptom control-based approach to an approach based on two essential aspects: remission (defined as a reduction/absence of symptoms to the point that they do not interfere significantly with behaviors) and functional improvement (defined as the ability to function, socially and vocationally, in the community) (37). In other words, recovery is a journey aimed at achieving a meaningful life which translates through an improved quality of life, physical health, social integration, instrumental competence and self-agency, and independent living (11). However, the low rate of recovery detected among individual with schizophrenia gives the idea that MHS have necessarily to further improve the current model of care by promoting the implementation of integrated and personalized treatments to overcome the existing barriers and, ultimately, to improve the functional outcome of affected individuals (4).

### 1.4 Background and study aims

Thus, we previously conduct a survey – co-designed by clinicians, expert patients and caregivers – with the aim to identify current unmet needs, gaps and limitations between current knowledges and clinical practice to help MHS to further organize an optimal journey for PLWS, throughout all the three phases of schizophrenia, with the ultimate goal to achieve recovery (35). This survey focused on the most populous Italian region, Lombardy (~9 million inhabitants), and analyzed the levels of importance and of implementation for several clinical actions/interventions or themes considered of significant importance by the panel of clinicians, patients’ and caregivers’ associations and expert patients (EXP patients). The survey examined three macro-areas corresponding to the three essential phases of the schizophrenia course, early detection and management, the acute phase management, and the long-term management/continuity of care. We found that, for the management of early phases, despite a great consensus on the actions to be implemented to treat young individuals was found, the degree of implementation in the real-life practice was only moderate-to-good. Considering the management of acute states of decompensation, strong consensus and a good level of implementation in clinical practice were found. Finally, considering the long-term management and the continuity of care, a strong consensus was found, but the level of everyday implementation was slightly moderately implemented. Overall, we observed that early phases and chronicity management have to be further implemented to improve the Patient Journey of PLWS (35).

Based on the results obtained at regional level, we aimed to extend the Patient Journey Project at national level to analyze the situation in Italian MHS and to understand the current areas of clinical intervention that are properly implemented and the areas that deserve more implementation to enhance the treatment plans for PLWS.

## 2 Materials and methods

### 2.1 Survey construction and survey aims

First, the scientific board (encompassing social researchers, psychologists and psychiatrists) was created to build the survey’s statements. The scientific board was as composed as we wanted to create a survey with a multidisciplinary approach, attentive to both clinicians’ and patients’ needs. This phase concerned a desk research design to review the existing Italian regulatory sources, guidelines and best practices on the management of mental frailties and schizophrenia (38–47). The scientific board identified three areas of interest: early detection and management, acute phase management, and long-term management/continuity of care, as they were considered the most significant areas in the ideal journey of PLWS. Then, according to the Italian regulatory sources, guidelines and best practices, the scientific board identified a list of possible statements and shared it with 8 representatives of 4 patients’ and caregivers’ associations (Coplotta, Diversamente, Anpis Puglia, Club Itaca Milano) and with 3 expert peer supporter patients (aka, ESP patients). ESP patients are patients diagnosed with schizophrenia according to the ongoing classification for mental disorders that are trained at the regional level through a dedicated class to be recognized as expert peer supporters. ESP patients and caregivers were included given their relevance to patient engagement and their empowerment in clinical and institutional settings (48). As we considered of strategic importance to include all the stakeholders involved in the ideal patient’s journey of PLWS, this phase of sharing was essential to reinforce our multidisciplinary approach to build the survey (49). To do so, a semi-structured one-on-one interview was conducted by one clinician and one social researcher with ESP patients and patients’ and caregivers’ associations, with the purpose of collecting real-life evidence and relating what had emerged from the guidelines and best practices with the unmet needs still present in the management of schizophrenia. From this interview, after a thorough validation process carried out by clinicians, ESP patients and caregivers, the scientific board codified the final sixty statements of the survey, focusing on peculiar themes and topics.

For early detection and management, we analyzed several themes including: services accessibility, continuity of care, multi-disciplinary evaluation of patients’ needs, rehabilitation, psychoeducational and psychotherapeutic interventions, and drug treatment’s safety and appropriateness. For acute phase management, we investigated the following topics: experience of hospitalization, prevention and decrease in commitment and forced treatment and physical restraints, and linkage to local and outpatient services. For long-term management/continuity of care, the topics were: individual treatment plans, psychoeducational interventions, continuity in drug treatment, patient’s physical health awareness, recovery and social integration interventions, social and job support, and residential and semi-residential interventions. The survey was deployed with the CAWI (computer-assisted web interviewing) method by using a web program created and developed to manage research, surveys and customer satisfaction studies. Finally, the survey comprised a 60-statements questionnaire built on the three main areas of interest, divided as follows (see, **Table 1**): 17 statements on the early detection and management, 16 statements on acute phase management, 27 statements on the long-term management/continuity of care. To answer the survey, the respondents had to express agreement or disagreement on a 5-point Likert scale. Each statement was analyzed according to 2 subscales. The first subscale assessed the importance of the statement, from (1) “of no importance” to (5) “extremely important”. The second subscale assessed the degree of implementation of the statement in the clinical practice, from (1) “not implemented at all” to (5) “extremely implemented”.

**Table 1:** Importance of statement and degree of implementation mean scores.

Statements		Importance	Implementation
Early detection and management			
1	Projects and protocols with child neuropsychiatry to promote access to adult psychiatric services	4.77	3.35
2	Projects and protocols with GPs aimed at prevention	4.03	2.77
3	Continuity of care between CAMHS and AMHS	4.68	3.6
4	Personalized project with continuous and intensive contacts in community MHS	4.78	3.75
5	Continuous and intensive contacts with family members	4.7	3.7
6	Multidisciplinary assessment of patient’s clinical and psychosocial problems	4.7	3.77
7	Using of internationally validated and widespread assessment tools	4.17	3.17
8	Assessment of family burden and their needs	4.6	3.6
9	Team-based multidisciplinary approach involving different healthcare professionals	4.87	3.88
10	Multidisciplinary support to family members	4.55	3.47
11	Home interventions	4.58	3.18
12	Psychotherapy	4.12	3.23
13	Psychoeducation	4.53	3.35
14	Rehabilitation	4.53	3.42
15	Work and study support interventions	4.6	3.23
16	Adequate pharmacological treatment for dosage and duration	4.67	4.12
17	Safety of pharmacological treatment	4.8	4.08
	*Total score*	4.57	3.51
Acute phase management			
18	Not necessary in acute inward admission	4.13	3.35
19	Improve accessibility to community MHS	4.68	3.62
20	Paying attention to emotive impact of hospitalization	4.52	3.57
21	Reduce involuntary admission	4.22	3.67
22	Avoid the use of physical restraint	4.65	3.88
23	Educational programs in order to minimize the need of physical restraint	4.68	3.87
24	Limit pharmacological restraint	4.08	3.33
25	Antipsychotic treatment as soon as possible	4.62	4.1
26	Minimum effective dosage	4.62	3.88
27	Safety of pharmacological treatment	4.77	4.17
28	Maintenance of pharmacological treatment for adequate time after discharge	4.3	3.82
29	Limit duration of hospitalization	4.1	3.7
30	Ensure rapid continuity of care with the community MHS	4.77	4.28
31	Intensive contacts with community MHS after discharge	4.65	3.87
32	Review of the treatment program during hospitalization among inpatient and outpatient healthcare professionals	4.58	3.7
33	Review of the treatment program between hospitalized patients and caregivers of the community mental health service	4.6	3.63
	*Total score*	4.5	3.78
Long-term management/continuity of care			
34	Continuous and multidisciplinary-based treatment	4.62	3.78
35	Define an Individual Treatment Plan identifying a Case Manager	4.57	3.73
36	Take care of the family members	4.47	3.37
37	Psychoeducational treatment for patients	4.57	3.35
38	Psychoeducational treatment for family members	4.5	3.28
39	Psychotherapeutic treatment for patients	4.42	3.12
40	Psychotherapeutic treatment for family members	3.98	2.68
41	Carefully managing substance abuse disorders with the help of Addiction Services	4.68	3.63
42	Monotherapy antipsychotic treatment	4.43	3.77
43	Clozapine in case of treatment-resistance	4.55	3.98
44	Evaluate physical health in collaboration with GPs	4.55	3.2
45	LAI treatment for patients with frequent relapses and poor adherence	4.62	4.1
46	Regular contacts with patients who stop drug treatment	4.55	3.38
47	Re-contact patients who interrupted the contact with the community MHS	4.47	3.45
48	Monitoring of patients’ lifestyle in collaboration with GPs	4.35	2.93
49	Peer support groups oriented to recovery and social inclusion	4.42	3.08
50	Integration of the Expert in peer support in multi-professional team	4.03	2.68
51	Role of the Expert in peer support in improving efficacy of treatments	4.03	2.62
52	Monitoring of adverse outcomes (death, suicide)	4.48	3.23
53	Assessment of patients’ job skills	4.45	3.62
54	Psychosocial interventions and work placement actions	4.6	3.52
55	Evidenced-based rehabilitation interventions either in community or Day-care facilities	4.43	3.68
56	Resocialization interventions either in community or Day-care facilities	4.42	3.8
57	Residential facilities in case of serious psychosocial functioning impairment	4.08	3.8
58	Rehabilitation programs in residential facilities in case of serious psychosocial functioning impairment	4.57	3.57
59	Rehabilitation programs in residential facilities aimed to patient’s return at home	4.68	3.55
60	Rehabilitation programs in semi-residential facilities for patients with a good level of autonomy	4.48	3.12
	*Total score*	4.44	3.41
	*Total mean score*	4.49	3.54

AMHS, adult mental health services; CAMHS, child and adolescence mental health services; GPs, general practitioners; LAI, Long-acting injectable antipsychotics; MHS, mental health services.

The survey was sent to Italian psychiatrists working as Heads of the Mental Health Departments and Addiction Services (MHDAS) or as facilities directors, regardless of whether they worked in academic or non-academic settings. No patients, caregivers or other stakeholders completed the survey.

Considering the study aims, the first purpose was to evaluate whether respondents could consider the selected statements to be of strategic importance according to their knowledge, best practice guidelines and national regulatory sources. This goal was achieved by analyzing the importance of the statement subscale. Subsequently, by analyzing the degree of implementation subscale, the survey aimed to evaluate whether the available knowledge and guidelines were currently implemented in clinical practice, according to the respondents’ judgment, and the possible *gaps* that exist between guidelines and clinical practice.

### 2.2 Statistical analyses

The results on the effective management of PLWS were analyzed considering a general overview of the responses, the assessment of the consensus level on the importance of the statement, the evaluation of the degree of implementation, the possible existing *gaps* between the guidelines and clinical practice.

Adopting only descriptive statistical analyses (mean scores, the mode and median values), the appropriate analyses were calculated using the IBM® SPSS Statistics Version 20 software. No *a priori* assumptions were made.

The interpretation of the results followed the same criteria that were applied in our previous publication (35) as follows:

- Importance of the statement subscale: a strong consensus was defined when rated as (4): “important” or above, whereas a poor consensus was defined when rated as (3) “quite important” or below. To quantify the consensus level on the importance of the statement, we derived a mean score for the three macro-areas of interest and a total score.
- Degree of implementation subscale: the results were reported by combining the degree of implementation in 3 groups according to the mean scores for each item in the three areas of interest. A good level of implementation was defined for a score rated as (4) “properly implemented” or above; moderate levels of implementation were rated as (3) “enough implemented”; and poor levels of implementation were rated as (2) “slightly implemented” or below. To quantify the degree of implementation, we derived a mean score for the three macro-areas of interest and a total score.
- The *gap* between the importance of the statement and the degree of implementation: this aspect was analyzed in order to identify the items that could benefit from further implementation through dedicated programs. The existence of the *gap* was defined when two conditions were satisfied: if the items of the importance of the statement subscale were above a score of 4, and if the items of the degree of implementation subscale underwent a score of 4. Thus, to define the existence of the *gap*, we implicitly considered the statements showing moderate levels of implementation, rated as (3) “enough implemented”.

For the interpretation of the results, we focused on the mean score values.

To strength our analyses, we also derived a *difference* (Δ) score by subtracting the mean values form the importance of the statement subscale to the degree of implementation subscale: when the Δ was bigger than 1, this meant that there was a large discrepancy between the importance attributed to the statement and the degree of implementation in the real-life context.

## 3 Results

### 3.1 Demographics

Respondents included 38 Heads of Mental Health Department and 22 facilities directors from all the Italian Regions, except for Basilicata, Friuli-Venezia Giulia, Sardinia and Veneto. Lombardy was the most represented Region (28.3% of the respondents), followed by Sicily and Campania (both, 13.3% of the respondents). More than half of the respondents (56.7%) worked in medium municipalities (10’000-100’000 inhabitants), 18.3% in medium-large municipalities (100’000-250’000 inhabitants), 15% in large municipalities (over 250’000 inhabitants) and 10% in small municipalities (under 10’000 inhabitants). No missing data was found, as all 60 respondents filled out all the statements.

### 3.2 Importance and implementation levels

The results on the importance of the statement and degree of implementation for the three macro-areas are summarized in **Table 1** (mean score values) and in **Table 2** (the mode and median values).

**Table 2:** Importance of statement and degree of implementation (mean scores, medians, mode and standard deviations).

Importance of statement				Degree of implementation		
	*Early detection and management*	*Acute phase*	*Long-term management*	*Early detection and management*	*Acute phase*	*Long-term management*
Mean	4.57	4.50	4.44	3.51	3.77	3.41
Standard deviation	0.65	0.76	0.71	0.94	0.91	1.03
Mode	5	5	5	4	4	4
Median	5	5	5	4	4	3

#### 3.2.1 Importance of the statements

Regarding the first subscale, assessing the importance of the statement, a strong consensus emerged for almost all statements of the survey. In the following section, for each macro-area, the first ten statements in order of importance are summarized. Considering the early detection and management, a strong consensus was found for all 17 statements. In detail, several items were considered of significant importance, especially on “to deliver a team-based multidisciplinary approach involving different healthcare professionals”, “promotion of projects and protocols with Child and Adolescent Mental Health Services (CAMHS) to promote and facilitate access to Adult Mental Health Services (AMHS)”, “to provide personalized projects with continuous and intensive contacts in community mental health services”, “to provide continuity of care between CAMHS and AMHS”, “to keep continuous and intensive contacts with family members”, “to provide a multidisciplinary assessment of patient’s clinical and psychosocial problems”, “to provide adequate pharmacological treatment for dosage and duration”, “to assess the family burden and their needs”, “to provide work and study support interventions in case of moderate/severe psychosocial functioning impairment”, and “to provide multidisciplinary support to family members”.

On the acute phase management, a strong consensus was found for all 16 statements. In particular, several items were considered of significant importance, especially on “to consider the safety of pharmacological treatment through an early monitoring of side effects”, “to ensure rapid continuity of care with community MHS”, “to improve accessibility to community mental health services”, “to organize educational programs to minimize the need of physical restraint”, “to avoid the use of physical restraint”, “to provide intensive contacts with community MHS after discharge”, “to set the antipsychotic treatment as soon as possible”, “to identify the minimum effective dosage”, “to review the treatment program between hospitalized patients and caregivers of the community mental health service”, and “to use APs at minimum effective dosage”.

In the long-term management/continuity of care, a strong consensus was obtained for 26 out 27 statements, with the exception on “to provide psychotherapeutic treatment for family members”. In detail, other items were considered of significant importance, especially on “to carefully assess and treat substance abuse disorders conjointly with dedicated addiction services”, “to provide rehabilitation programs in residential facilities aiming for the patient’s return at home”, “to provide continuous and multidisciplinary-based treatment to promote full psychosocial recovery”, “to offer LAI antipsychotic treatment in case of frequent relapses and poor adherence”, “to provide psychosocial interventions and work placement support”, “to define an individual treatment plan and to identify a case manager”, “to provide psychoeducational treatments for patients”, “to provide rehabilitation programs in residential facilities in case of serious psychosocial functioning impairment”, “to offer clozapine in case of treatment-resistance”, “to evaluate physical health in collaboration with GPs” and “to maintain regular contacts with patients who stop drug treatment”.

#### 3.2.2 Degree of implementation

**Figure 1:**
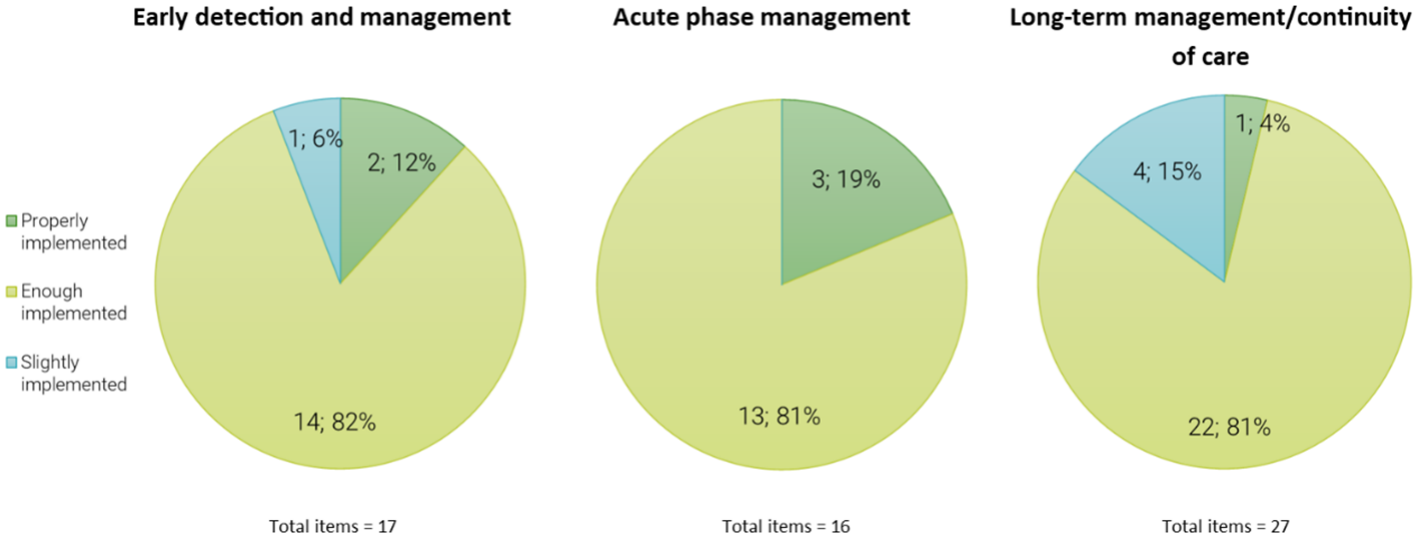
Degrees of implementation. The figure shows the number of items (and the percentage of the total items for each of the three thematic areas) divided according to the level of implementation. The results of the degree of implementation subscale are subdivided into 3 groups according to the mean scores for each item in the three areas of interest. Good level of implementation was defined for a score rated as (4) “properly implemented” or above; moderate levels of implementation was rated as (3) “enough implemented”; poor levels of implementation was rated as (2) “slightly implemented” or below.

The second subscale assessed the degree of implementation. **Figure 1** offers a general overview of the levels of implementation among the three thematic areas reporting the total items and the percentage for each level of implementation on an individual thematic area.

Good levels of implementation were found on 2 out of 17 statements (12% of the sample) for the early detection and management area, particularly on “to provide adequate pharmacological treatment for dosage and duration” and “to consider the safety of pharmacological treatment”. Good levels of implementation were found on 3 out of 16 statements for the acute phase management area (19% of the sample), particularly on “to ensure a continuity of care with community MHS”, “to consider the safety of pharmacological treatment”, and “to start as soon as possible an antipsychotic treatment”. Good levels of implementation were found on 1 out of 27 statements for the long-term management/continuity of care area (4% of the sample), particularly on “to offer LAI treatment in case of frequent relapses and poor adherence”.

Poor levels of implementation were found on 1 out of 17 statements of early detection and management (6% of the sample) (i.e., “to promote projects and protocols with GPs aimed at prevention”), none of acute phase management, and 4 out of 27 statements on the long-term management/continuity of care (15%) (i.e., “to monitor patients’ life-style in collaboration with GPs”, “to promote the integration of the Expert in peer support in multi-professional team”, “to provide psychotherapeutic treatment for family members” and on the “role of the Expert in peer support in improving efficacy of treatments”).

#### 3.2.3 The gap of the statement: importance vs degree of implementation

We measured the *gap* between the importance of the statement and the degree of implementation by considering the statements with a moderate level of implementation.

**Figure 2:**
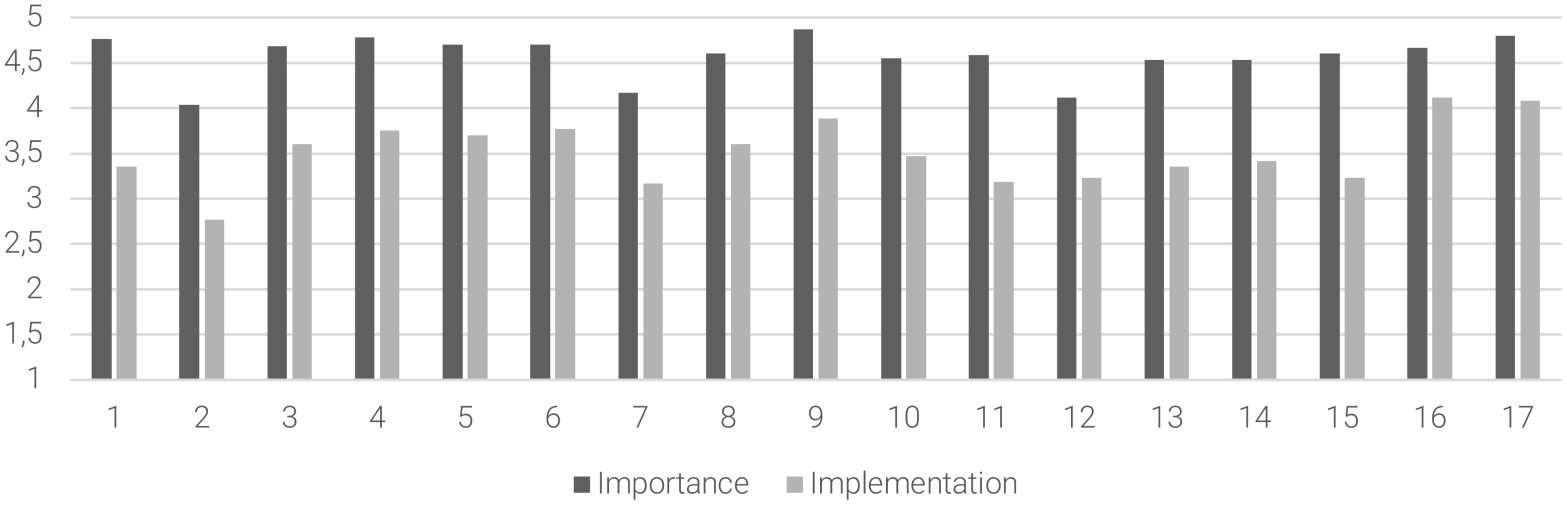
The gap between the importance of the statement and the degree of implementation in the early detection and management area. Abscissa axis describes the items (1–17) of the early detection and management area. Ordinate axis represents the 5-point Likert scale anchor points: deep gray for importance of the statement (from (1) “of no importance” to (5) “extremely important”) and light gray for degree of implementation (from (1) “not implemented at all” to (5) “extremely implemented”).

For the early detection and management, moderate levels of implementation were found on 14 out of 17 statements (82% of the sample), especially on “to deliver a team-based multidisciplinary approach involving different healthcare professionals”, the “to provide a multidisciplinary assessment of patient’s clinical and psychosocial problems”, “to create a personalized project with continuous and intensive contacts in community mental health services”, “to keep continuous and intensive contacts with family members”, “to provide continuity of care between CAMHS and AMHS”, and “to assess the family burden and their needs”. For the early detection and management, **Figure 2** summarizes the mean scores of the importance of the statement and degree of implementation subscales and the *gap* between these subscales.

**Figure 3:**
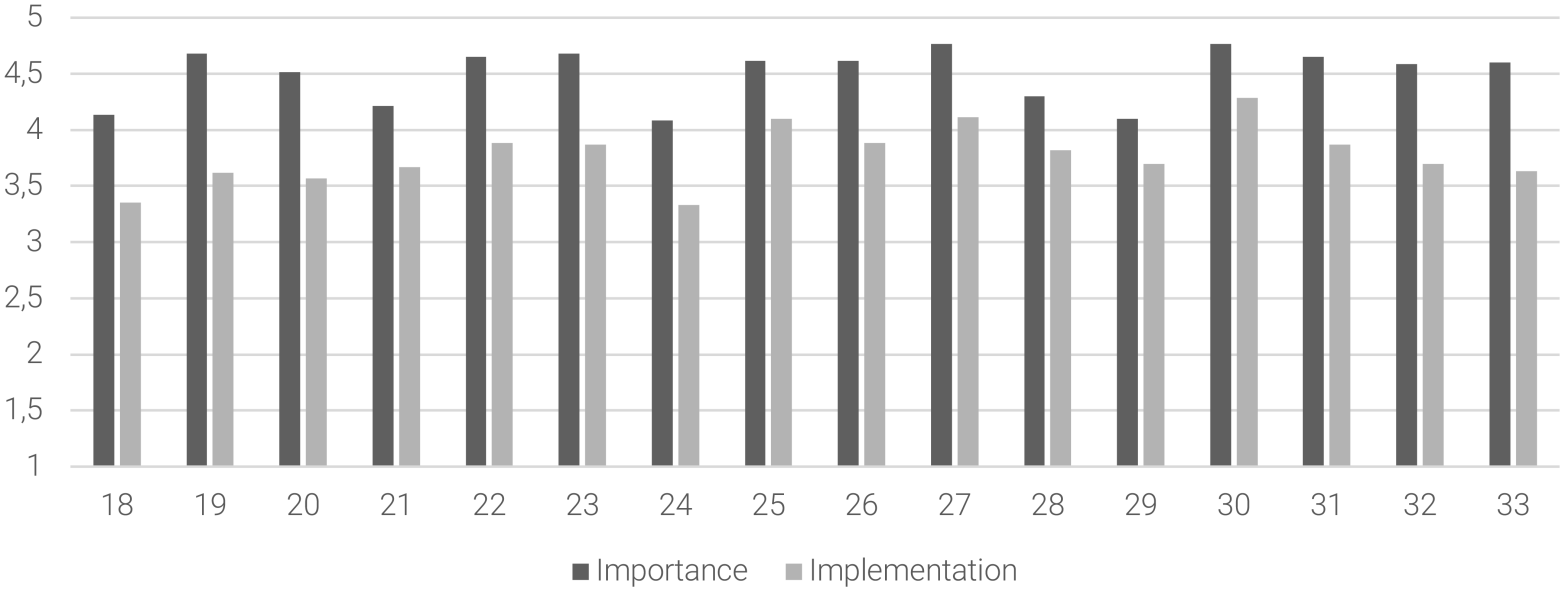
The gap between the importance of the statement and the degree of implementation in the acute phase management area. Abscissa axis describes the items (18–33) of the acute phase management area. Ordinate axis represents the 5-point Likert scale anchor points: deep gray for importance of the statement (from (1) “of no importance” to (5) “extremely important”) and light gray for degree of implementation (from (1) “not implemented at all” to (5) “extremely implemented”).

Considering the acute phase management, moderate levels of implementation were found on 13 out of 16 statements (81% of the sample), especially on “to avoid the use of physical restraint”, “to identify the minimum effective dosage”, “to organize educational programs in order to minimize the need of physical restraint”, “to ensure intensive contacts with community MHS after discharge”, “maintenance of pharmacological treatment for adequate time after discharge”, “to limit the duration of hospitalization”, “to review the ongoing treatment plans, when an hospitalization occurs, through a collaboration between inpatient and outpatient healthcare services”, “to reduce involuntary admission”, “to review the treatment program between hospitalized patients and caregivers of the community MHS” and “to improve accessibility to community MHS”. **Figure 3** summarizes the mean scores of the importance of the statement, degree of implementation, and the *gap* between these subscales for the items related to the acute phase management area.

**Figure 4:**
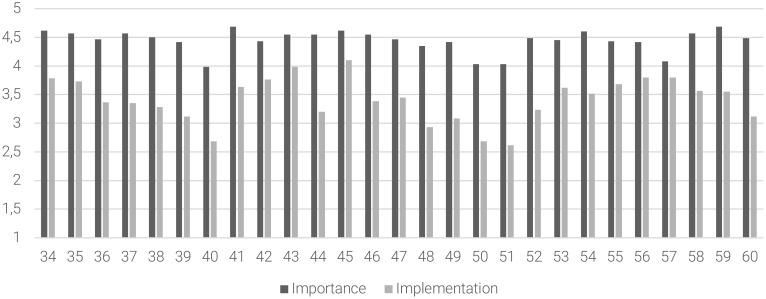
The gap between the importance of the statement and the degree of implementation in the long-term management/continuity of care area. Abscissa axis describes the items (34–60) of the long-term management/continuity area. Ordinate axis represents the 5-point Likert scale anchor points: deep gray for importance of the statement (from (1) “of no importance” to (5) “extremely important”) and light gray for degree of implementation (from (1) “not implemented at all” to (5) “extremely implemented”).

Concerning the long-term management/continuity of care, moderate levels of implementation were found on 22 out of 27 statements (81% of the sample), especially on “to offer clozapine in case of treatment-resistance”, “to offer resocialization interventions either in community and/or Day-care facilities”, the “need of residential facilities in case of serious psychosocial functioning impairment”, “to provide continuous and multidisciplinary-based treatment to promote full psychosocial recovery”, “to define an Individual Treatment Plan and to identify a case manager”, “to provide monotherapy antipsychotic treatment”, “to provide evidence-based rehabilitation interventions either in community and/or Day-care facilities”, “to carefully assess and treat substance abuse disorders conjointly with dedicated addiction services”, “to evaluate physical health in collaboration with GPs” and “to assess the patients’ job skills in the Individual Treatment Plan”, and “to provide psychosocial interventions and work placement actions”. **Figure 4** summarizes the mean scores of the importance of the statement and the degree of implementation subscales and the *gap* between these subscales for the long-term management/continuity of care.

**Figure 5:**
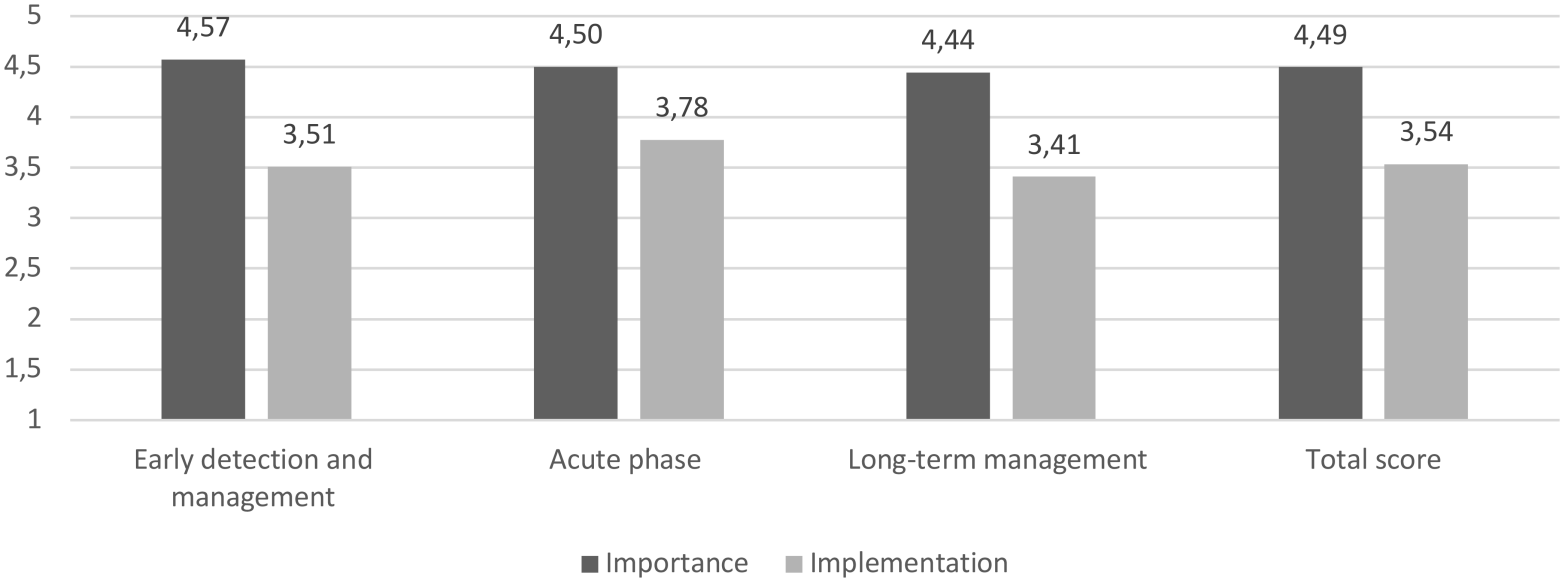
The *gap* between the importance of the statement and the degree of implementation (mean scores) for the three macro-areas of interest and the total score. Deep gray for the importance of the statement subscale (from (1) “of no importance” to (5) “extremely important”); light gray for the degree of implementation subscale (from (1) “not implemented at all” to (5) “extremely implemented”).

Considering the overall results from the three macro-areas, the survey found a strong consensus (mean score = 4.49) and a moderate level of implementation (mean score = 3.54) for the analyzed statements. More in detail, for the early diagnosis and management, while a strong consensus was found regarding the importance of the statements (mean score = 4.57), the level of implementation in the real-world practice was found to be moderate (mean score = 3.51). For the acute phase management, the survey revealed a strong consensus (mean score = 4.50) and a moderate level of implementation (mean score = 3.78). For the long-term management/continuity of care, while a strong consensus was found regarding the importance of the statements (mean score = 4.44), the implementation in the real-world practice was found to be at moderate level (mean score = 3.41). Particularly, the implementation level for the long-term management/continuity of care was the lowest among the three macro-areas. **Figure 5** summarized the overall mean scores of importance of the statement, degree of implementation, for the three macro-areas of interest.

#### 3.2.4 The difference (Δ) between the importance of the statement subscale and the degree of implementation subscale

**Figure 6:**
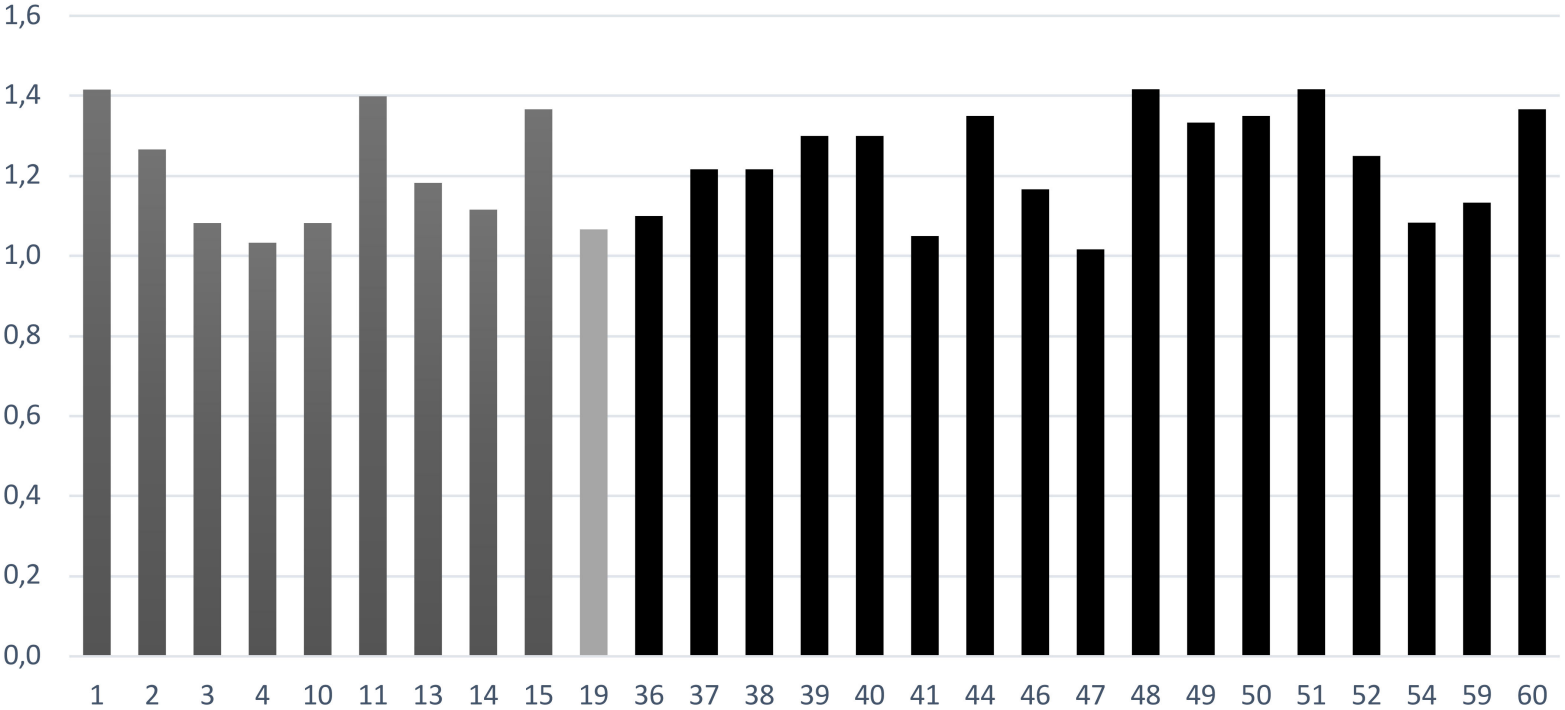
The statements for which importance of the statement subscale and degree of implementation subscale have a difference **Δ** bigger than 1. Deep gray for early diagnosis and management, light gray for acute phase management and black for long-term management/continuity of care.

Results are described in **Table 3** and **Figure 6**. Considering the entire survey, several statements (27 out of 60) presented a Δ between importance of the statement and degree of implementation. In particular, for early diagnosis and management, 9 items out of 17 showed a Δ bigger than 1, especially “to provide projects and protocols with CAMHS to promote and facilitate access to AMHS”, “to deliver home interventions”, “to provide work and study support interventions in case of moderate/severe psychosocial functioning impairment” and “to promote projects and protocols with GPs aimed at prevention”. For the acute phase management, 1 item out of 16 showed a Δ bigger than 1 (“to improve accessibility to community mental health services”). For the long-term management/continuity of care, 17 items out of 27 showed a Δ bigger than 1, especially “to regularly monitor patients’ life-style in collaboration with GPs”, “to promote the role of the expert in peer support in improving efficacy of treatments”, “to promote rehabilitation programs in semi-residential facilities for patients with a good level of autonomy”, “to promote the integration of the Expert in peer support in multi-professional team”, “to regularly monitor patients’ physical health in collaboration with GPs”, “to promote the participation to peer support groups oriented to recovery and social inclusion”, “to provide psychotherapeutic treatment for patients”, “to provide psychotherapeutic treatment for family members”.

**Table 3:** The difference (Δ, bigger than 1), between the importance of the statement and the degree of implementation.

Item	Statement	Δ
Early detection and management		
1	Projects and protocols with child neuropsychiatry to promote access to adult psychiatric services	1.42
11	Home interventions	1.40
15	Work and study support interventions	1.37
2	Projects and protocols with GPs aimed at prevention	1.27
13	Psychoeducation	1.18
14	Rehabilitation	1.12
3	Continuity of care between CAMHS and AMHS	1.08
10	Multidisciplinary support to family members	1.08
4	Personalized project with continuous and intensive contacts in community mental health services	1.03
Acute phase management		
19	Improve accessibility to community MHS	1.07
Long-term management/continuity of care		
48	Monitoring of patients’ lifestyle in collaboration with GPs	1.42
51	Role of the Expert in peer support in improving efficacy of treatments	1.42
60	Rehabilitation programs in semi-residential facilities for patients with a good level of autonomy	1.37
50	Integration of the Expert in peer support in multi-professional team	1.35
44	Evaluate physical health in collaboration with GPs	1.35
49	Peer support groups oriented to recovery and social inclusion	1.33
39	Psychotherapeutic treatment for patients	1.30
40	Psychotherapeutic treatment for family members	1.30
52	Monitoring of adverse outcomes (death, suicide)	1.25
37	Psychoeducational treatment for patients	1.22
38	Psychoeducational treatment for family members	1.22
46	Regular contacts with patients who stop drug treatment	1.17
59	Rehabilitation programs in residential facilities aimed to patient’s return at home	1.13
36	Take care of the family members	1.10
54	Psychosocial interventions and work placement actions	1.08
41	Carefully managing substance abuse disorders with the help of Addiction Services	1.05
47	Re-contact patients who interrupted the contact with the community MHS	1.02

## 4 Discussion

The present survey was aimed to elucidate the current situation on the value of Patient Journey for PLWS taking in consideration the perspective of Heads of MHD and facilities directors around Italy. Indeed, this study explored the strengths and weaknesses of Italian MHS interventions for PLWS: the idea was to highlight the existing barriers to be addressed if MHS want to organize an optimal treatment plan for PLWS to further improve quality of life and to achieve full recovery. Studies like this are important to provide an overview, at a national level, on the care of PLWS as the national MHS situation may indirectly reflect what happens at a regional level, where the organization of local MHS could substantially differ due to inter-regional difference in resources allocation (especially for what concerns the access and intensity of psychosocial care) (28, 50). The present survey investigated the point of view of Heads of MHD and facilities directors to understand the realistic obstacles and unmet needs that clinicians must face in the everyday practice to improve the care management of PLWS. Furthermore, a strength of the present work is that the survey was co-designed with the contribution of all the stakeholders involved in the ideal path of care of PLWS (including clinicians, expert patients, caregivers and family associations) in order to provide a multidisciplinary assessment of the current situation among Italian MHS. Doing so, we have analyzed three macro-areas (early diagnosis and management, acute phase management and long-term management/continuity of care) as these phases are crucial steps during the insidious course of schizophrenia. Thus, we discussed the consensus on the importance of several areas of intervention and the existing gap between the importance of the statements and their implementation among Italian MHS, also analyzing the areas presenting with good, poor and moderate levels of implementation.

### 4.1 The consensus level on the importance of the statements

Considering results from the importance of the statement subscale, the fact that a strong consensus was reached for almost the statements suggests that Heads of MHDAS and facilities directors have clearly in mind that the management of the ideal Patient Journey for PLWS must involve all the phases of schizophrenia, from the early stages of detection and treatment up to the management of acute decompensation and, above all, to the management of chronicity. These results are congruent with National guidelines (46, 47) and international literature (11) suggesting that differential clinical actions should be organized according to the different clinical needs that PLWS could present throughout the course of their illness. Conversely, respondents reached a poor consensus on only one item in the long-term management/continuity of care area that examined the provision of psychotherapeutic treatment for family members. Moreover, this statement also showed a poor level of implementation. Although the family milieu has a central role in caring for PLWS with an increased emotional cost for caregivers (51), we found that family interventions are poorly implemented in clinical practice despite their strong scientific basis (52, 53).

These finding have important implications clinically: in fact, on the one hand they suggest the need to further raise awareness among Heads of MHDAS in promoting psychotherapeutic interventions for PLWS and for their families. Furthermore, since scientific evidence has found robust benefits for psychotherapeutic treatments (including, family interventions, family psychoeducation, and cognitive behavioral therapy) in preventing relapse (52) and reducing symptoms burden (53) both in the early phases (53) and in the long-term treatment (52), we recommend that national providers and legislators will provide specific plans to further implement these neglected interventions in clinical practice.

### 4.2 Good levels of implementation in clinical practice: the role of pharmacological treatments

The survey also found that only a minority of the statements showed good levels of implementation. Particularly, 2/17 statements in the early detection and management, 3/16 statements in the acute phase management and 1/27 statement in the long-term management/continuity of care reached good levels of implementation. This was especially true for the statements that examined the role of pharmacological treatments throughout the course of schizophrenia. Indeed, it was found that respondents considered that the provision of an effective and well-tolerated antipsychotic treatment is among the few aspects already implemented in clinical practice. Indeed, for the early detection and management area, two statements presented good level of implementation (to provide adequate pharmacological treatments and to consider the safety and tolerability profiles of AP compounds): these results are in line with a recent Delphi study on the pattern of care for adolescents with schizophrenia, suggesting that SGA should be preferred to FGA as they are associated to better tolerability and safety profile (54). Moreover, this confirms that the idea that the choice of the AP compound by clinicians should be guided by the subjective characteristics of the affected individual (including, the symptomatic manifestations, the illness stage, the presence of medical comorbidities or concomitant substance abuse) and should be shared with the patient and the family (54). Similarly, in the acute phase management area, good levels of implementation were found on the statements that consider the provision of a safe and well-tolerated pharmacological treatment with the idea to start as soon as possible an AP prescription. Thus, given the documented efficacy of different AP during acute states of decompensation (55), it must be highlighted that clinicians should personalize AP prescription taking into consideration the balancing between the effectiveness of a given molecule and possible related side effects (56). Furthermore, as the milestone in treating of PLWS is to achieve functional recovery and better quality of life, it is not surprising that the survey found that, in the long-term management/continuity of care, the possibility to offer LAI treatment in case of frequent relapses and poor adherence was at good level of implementation. This confirm that clinicians considered LAI regimen an essential strategy to improve adherence and prevent the risk of relapse and of new hospitalizations (33, 33).

These findings have important clinical implications especially for what concerns the personalization of pharmacological treatments: what could guide the clinician in personalizing the treatment, through the process of shared decision-making (34), is a careful evaluation of the tolerability profile of the chosen molecule for example by taking into consideration the anticholinergic burden and the metabolic effects that characterize each antipsychotic compound.

### 4.3 Poor levels of implementation in clinical practice: the role of GPs and ESP patients

On the other hand, the survey also to demonstrate that, among Italian MHS, some interventions had poor levels of implementation: in detail, in 1/17 statements of early detection and management and 4/27 statements on the long-term management/continuity of care, but none in the acute phase management. These results are somewhat encouraging as they reveal how the Italian Heads of MHDAS have been able to adequately implement most of the areas of intervention throughout the journey of PLWS. Nevertheless, it’s important to highlighted that, both in the early detection and long-term management, an issue that deserves further implementation is the effective involvement of GPs throughout the schizophrenia course, especially in preventive actions to detect individual in prodromal/early phases and in the management of chronicity evaluating patients’ lifestyle and physical health. As PLWS have more physical problems than the general population, GPs have an essential role in the management of mental and physical comorbidity as they often are the main entry point into the healthcare system (57). Moreover, GPs give support and information to the patient’s family (57). Thus, we recommend to further promote, through specific awareness campaigns, the collaboration between GPs and MHDAS to improve quality of care for PLWS especially by monitoring lifestyle and physical health.

Another poorly implemented topic in the long-term management area was the role of ESP patients in peer support interventions. Based on the idea that people who have experienced mental disorders can provide care to others dealing with similar problems (58), peer support programs performed by ESP patients could help other individuals to become more active in their own process of recovery (58). Although some evidence suggested that peer support interventions could exert an effective role improving clinical (i.e., acute care utilization, positive and negative symptoms) (59) and functional outcomes (i.e. recovery) (60), other findings noted that limited data are available to definitively recommend peer support for PLWS (61). Despite these controversial results, as peer support is an essential component of the PLWS journey improving the autonomy and the participation in treatment decision, we deem that MHDAS should further improve this intervention in clinical practice with dedicated plans and awareness campaigns.

### 4.4 Moderate levels of implementation in clinical practice

The most important result from the survey is that majority of interventions for PLWS were found to be moderately implemented in clinical practice among Italian MHDAS. Indeed, for all the three macro-areas, the implementation levels were moderate, with the highest implementation for the acute phase management and the lowest for long-term management/continuity of care. These results could be interpreted in two different ways: on the one hand, we could suggest that the implementation levels are more than acceptable among Italian MHS. On the other hand, we can observe that clinical practice still requires further strengthening to effectively improve the journey of PLWS as many areas and interventions present some *gaps* between the importance of the statements and the implementation. As the level of importance for the statements is high for all the investigated areas, we can hypothesize that the heterogeneous implementation levels in clinical practice could be principally attributable to the inter-regional differences in resources allocation and availability that are responsible of the different organization of mental health care at local level. However, this is only a suggestion as this study did not systematically investigate inter-regional differences between local MHS. Moreover, no other studies are available to compare our results, except for our previous survey that examined the Lombardy MHDAS (35). In this case, it was found that, for the early detection and long-term management areas, the degree of implementation in the real-life practice was only moderate-to-good and slightly moderate, respectively, while for the acute phase management, good levels of implementation were found. These data lead to conclude that early phases and chronicity management were the main areas requiring further implementation to improve the journey of PLWS (35). Thus, we can observe that the results of the present survey are substantially in line with those of our previous investigation, with the exception of the acute phase management area.

As moderate levels of implementation emerged for several items, in the following paragraphs we delve into the main areas that could benefit from greater empowerment of clinical actions.

#### 4.4.1 Moderate levels of implementation in the early detection and management phase

We found that 14/17 statements had moderate levels of implementation. In line with our previous survey (35), current findings suggest that the early detection and management is an essential area that must be further improved to promote an effective patient’s journey. The statements at moderate level of implementation covered an important topic that is the need to provide continuity of care between CAMHS and AMHS. Despite MHS need to appropriately respond to the adolescent population transitioning from the CAMHS to AMHS, the scenario in Italian MHS is substantially jeopardized. Indeed, in Italy, two operational models exist to manage the individuals during the early stages of the disorder (54). The first model is based on the guidance of the National Action Plan for Mental Health (62) that recommended to develop multidisciplinary teams involving CAMHS and AMHS together with families, educational facilities and the environmental context in order to develop projects aimed at prevention and early intervention. The second approach is based on international evidence on early intervention programs for psychosis according to the Clinical High risk of psychosis (CHR-P) model (63) that suggested to create a transitional team operating independently from youth and adult services. In Italy, the ITAlian Partnership for Psychosis Prevention (ITAPP) project was created including five CHR-P academic centers across Italy (Pavia, Milan, Naples, Bari, Perugia), established from 2007 to 2018, and it was aimed at early detection and intervention. Serving adolescents and young adults with multidisciplinary and integrated interventions, ITAPP project offered the possibility to develop specialized facilities bridging the gap in the transitioning phase from CAMHS to AMHS, reducing the duration of untreated psychosis and ameliorating clinical symptoms. Nevertheless, it should be noted that main weakness of ITAPP model is to introduce an additional split within the MHS (64).

In the early detection and management area, a critical issue deserving further implementation is the creation of an individualized project with continuous contacts in the community MHS. This individualized project should involve the provision of a team-based multidisciplinary approach able to assess the patient’s clinical and psychosocial problems, also taking in consideration family burdens and encouraging home/work/study support interventions, psychoeducation and rehabilitative plans. Congruently, another topic at moderate levels of implementation was to provide a multidisciplinary assessment of patient’s clinical and psychosocial problems. However, recent findings noted that, in Italy, a structured assessment of clinical and psychosocial problems was infrequent especially for newly taken-in-care patients (28). These recommendations were already be highlighted in the early 2014 in the *Definizione dei percorsi di cura da attivare nei Dipartimenti di Salute Mentale per i Disturbi schizofrenici, i Disturbi dell’umore e i Disturbi gravi di personalità* agenda (46).

The fact that the implementation of early intervention services, by using structured clinical pathways for newly taken-in-care patients, is only moderately realized leads us to recommend that MHS should further promote individualized programs with multidisciplinary actions to improve the treatment of PLWS at early stages. Moreover, although there is not a definitive model for an effective organization of MHS for young people in early stages of the disorder, this recommendation is in line with a recent Italian study that highlighted the need to increase the number of outpatient services aimed at the early detection and intervention for help seeker individuals (4).

#### 4.4.2 Moderate levels of implementation in the acute phase management

We found that 13/16 statements were at moderate levels of implementation in this phase. These statements covered several topics, including to limit the duration of hospitalization and to reduce involuntary admission but also to review the therapeutic program between hospitalized and outpatient services/caregivers and to maintain pharmacological treatment for adequate time after discharge. All of these clinical actions were already recommended in the *Definizione dei percorsi di cura da attivare nei Dipartimenti di Salute Mentale per i Disturbi schizofrenici, i Disturbi dell’umore e i Disturbi gravi di personalità* agenda (46). Once again, the fact that all these statements are recognized as of primary importance, despite their moderate level of implementation, leads us to recommend that MHS must necessarily implement their efforts to better manage all of these topics during the acute state of decompensation phase.

Other important topics at moderate level of implementation were to ensure intensive contacts with outpatient services after discharge and to improve accessibility to community MHS. A clinical implication of these findings leads us to consider the need to implement outpatient services: as the continuity of care between inpatient and community services is a critical element to improve the journey of PLWS after a phase of acute decompensation, our findings suggest that the hospital-community relationship should be extensively reconfigured. In fact, it was demonstrated that, in community-based MHS, the length of stay and the risk of rehospitalization are lower compared to the hospital-based systems (65). This data confirms the idea that the management of the acute phase of decompensation could take place in different settings (for example, outpatients services, semi-residential facilities or with home care interventions) and should not be limited to the hospitalization. Thus, to effectively enhance continuity of care, we recommend that MHS should guarantee a stronger regulation of care provision and financing with the aim to implement the provision of outpatient services at a local level (66). Anyway, recent findings suggested that the actual scenario on service utilization during an acute crisis is not so dramatic in Italy. Employing data from healthcare utilization databases, Lora and colleagues found that only a minority of PLWS had a hospitalization lasting more than 30 days and that 1/5 cases of the acute admissions were followed by readmission within 30 days of discharge (28). Moreover, they found that continuity of care between acute inward and outpatient services (i.e. at least one outpatients service contact within 14 days following acute inward units discharge) was achieved for 6/10 cases after discharges (28), albeit home care (within 2 weeks after hospital admission) was provided only for 1/20 cases in chronic patients after discharge. However, for new taken-in-care patients, home visits were even rarer (28). Thus, we can therefore hypothesize that the discrepancies between the results of our survey and the results from Lora and colleagues are linked to methodological differences within the studies: in fact, the present survey is based on the point of view of the Heads of the MHDAS while Lora’s results are based on the analysis of the healthcare utilization databases in four Italian regions (Lombardy, Emilia-Romagna, Lazio, Sicily) (28).

Other topics found at moderate levels of implementation in the acute phase management were to avoid the use of physical restraint and the need to organize educational programs to minimize physical restraint. These points were already highlighted in our previous survey for Lombardy MHS (35). Although it is not feasible to completely abolish events of physical restraint as these are influenced by psychopathological manifestations (i.e., the severity of positive and disorganization symptoms) (67), by sociodemographic factors (being male, at younger age) (68, 69) and by the type of admission (e.g., compulsory hospitalization) (70), metanalytic evidence suggested that the delivery of educational programs for mental health workers, the use of physical restraint is significantly reduced (71). This is especially true if education programs involve nurses and are continuously provided over time (72). Concluding, although no definitive strategies are available to reduce risk of physical restraint, a recommendation is that MHS proactively implement educational interventions for acute inward units to reduce episodes of physical restraint.

#### 4.4.3 Moderate levels of implementation in the long-term management/continuity of care

Moderate levels of implementation were found on 22/27 statements. These statements covered some important topics in clinical practice, including to provide continuous multidisciplinary treatments to promote full psychosocial recovery, to offer resocialization interventions either in community or semi-residential facilities, to engage PLWS in residential facilities in case of serious psychosocial functioning impairment, to provide evidence-based rehabilitation interventions both in community and in semi-residential facilities and to provide psychosocial interventions and work placement actions and, at least, to assess the patients’ job skills. These results are in line with other findings that observed how in Italy the delivery of psychosocial interventions is not satisfying: Lora and colleagues found that home care, psychoeducational and psychological treatments for both PLWS and family members are not commonly provided, and the intensity of these treatments is only moderate (28). Detailly, the Authors found that 1/10 patient had access to psychological interventions/psychoeducation and that activities addressed to families involved only a third of chronic patients (28). Moreover, 1/6 patient was admitted to a community residential facility (28). Although all these interventions were considered of primary importance in the “Definizione dei percorsi di cura da attivare nei Dipartimenti di Salute Mentale per i Disturbi schizofrenici, i Disturbi dell’umore e i Disturbi gravi di personalità” agenda (46), results from our survey confirmed the idea that community care for PLWS, especially in case of chronicity, is more focused on providing psychiatric care rather than on psychosocial care. This fact means that the current therapeutic approach in the management of chronicity is, more often than not, stereotyped and not focused on an effective personalization and integration of pharmacological and psychosocial treatments to obtain a full recovery. The clinical impact of these results leads us to conclude that an urgent need for the Italian MHS is to effectively improve the provision of community psychosocial cares to further increase the management during chronic phases. As suggested by Lora and colleagues, a recommendation is to empower nurses, rehabilitation therapists and social workers as feasible providers of psychosocial interventions through a wide and structural process of task shifting (28). We are in fact convinced that, by doing so, MHS will be able to realize the key principle of chronicity management, namely the creation of multidisciplinary community-based interventions that is expressed in the definition of the so-called individual treatment plan and in the application of an efficient case management approach. In fact, the survey found that the realization of an individual treatment plan, through a case management approach, is only at moderate level of implementation. In this scenario, a suggestion could be to potentiate the role of semi-residential facilities that are designed to solve a therapeutic-rehabilitative action in the community context counterbalancing the phenomenon of interminable psychiatric residency by promoting rehabilitation, socialization and social reintegration (35, 46). Moreover, as the efficacy of several psychosocial interventions (i.e., cognitive rehabilitation, social skill training, cognitive behavioral therapy, supported employment, family intervention and psychoeducation) (73–76) was clearly established by several scientific evidence, we recommend that MHS will systematically integrate these evidence-based psychosocial approaches in clinical practice to improve the management of PLWS during chronic phases. Therefore, we believe that it is necessary for the clinicians to improve their training on the application of these psychosocial interventions through specific educational and academic campaigns. Nonetheless, if MHSs are to become truly recovery-oriented, we believe that a profound review of the allocation of human and economic resources is essential to systematically implement these psychosocial interventions in clinical practice.

Despite both statements reached a strong consensus by survey respondents, moderate levels of implementation were found for the provision of clozapine in case of treatment-resistance schizophrenia and for the provision of antipsychotic monotherapy regimen. We deem necessary to remember two important issues. On one hand, as non-adherence phenomenon is strongly associated with polypharmacy (29, 30), clinicians should consider to revise AP prescription favoring a monotherapy regimen with the aim to reduce the risk of drug discontinuation and subsequent relapse. On the other hand, the scientific evidence on clozapine efficacy, if compared to FGAs and SGAs, in case of refractory cases is quite massive despite its unfavorable metabolic and hematological profile (77). For these reasons, we recommend that clinicians should not fear using clozapine in cases of resistant schizophrenia as already suggested in the “Definizione dei percorsi di cura da attivare nei Dipartimenti di Salute Mentale per i Disturbi schizofrenici, i Disturbi dell’umore e i Disturbi gravi di personalità” agenda (46).

Moreover, as the management of concomitant substance misuse was found to be only moderately implemented in long-term management/continuity of care, some actions could be recommended to be implemented the clinical practice. On the one hand, a feasible option is to provide LAI antipsychotics regimen as several evidence demonstrated it efficacy in treatment comorbid substance abuse in PLWS (78–80). On the other hand, meta-analytic evidence suggested that clozapine was superior to other AP in reducing substance misuse (81), while other findings observed that clozapine is strongly associated with reduced risk of developing substance use disorders among PLWS (82) maintaining them abstinent from misuse (83).

## 5 Limitations

Our study has some limitations. Although the respondents to our survey represent a good sample including the majority of Italian regions, we must note that results from some highly populated regions (including Veneto, Friuli-Venezia Giulia, Sardinia and Basilicata) are missing. This may have led to underestimation of our results. Furthermore, more than half of the respondents worked in medium-sized municipalities (10,000-100,000 inhabitants), while data from larger municipalities is limited. This may have led to bias our results: further examination should analyze the data by dividing it according to the size of the municipality. This type of analysis would allow us to observe whether there are differences in implementation levels depending not only on geographical areas, but above all, on the size of the municipality. This could further clarify whether MHS are differentially implemented in smaller or larger cities.

We recognize that another possible limitation is linked to the fact that data were exclusively collected through a self-report survey, leading to possible response bias: indeed, participants might have provided socially desirable responses or inaccurately reported their practices, leading to biased results.

## 6 Conclusions

This survey, co-designed by clinicians, expert patients and caregivers, offered an updated evaluation of the interventional areas considered at priority importance for the Heads of MHD and facilities directors to formulate an effective journey for PLWS that will consider three main phases in the course of schizophrenia. Additionally, the survey highlighted several unmet needs to the actual implementation of the journey of PLWS by analyzing the areas at good, moderate and poor level of implementation in clinical practice. While the respondents clearly perceived that it is necessary to formulate specific clinical actions for each stage during the course of the disorder, the most important result is that majority of interventions for PLWS were found to be moderately implemented in clinical practice in Italian MHDAS. Among these areas, the topics that deserve most implementation included the interventions during the early stage (especially the continuity of care between CAMHS and AMHS), the need to provide evidence-based psychosocial interventions during the chronic stages of the disorder and the need to assure continuity of care after acute hospitalization. Moreover, other areas that deserve further implementation though dedicated plans are the involvement of GPs and of EXP patients in the care of PLWS. On the other hand, clinicians seem to have clearly in mind the importance of providing personalized pharmacological interventions throughout all phases of the schizophrenia as this aspect was found al good level of implementation. Thus, Italian MHS have to provide new strategies and structural actions to overcome these current limitations and effectively improve the journey of PLWS. In line with previous suggestions by Galderisi and colleagues (4), the results of the present investigation suggest that mental health professionals and national legislators need to improve awareness of the urgent need to provide integrated and personalized treatments (i.e., effective pharmacotherapy and well tolerated, physical health monitoring and early intervention plans) to further improve current clinical practice. Sharing with Galderisi and colleagues the idea that the main barriers to the effective implementation of treatment paths are linked to the lack of time, human and financial resources, as well as adequate training (especially with regard to the application of psychosocial interventions based on the evidence) (4), we believe it is necessary for national legislators to address these structural problems in order to allow MHS to become effectively, and not just in theory, recovery-oriented.

## Data availability statement

The raw data supporting the conclusions of this article will be made available by the authors, without undue reservation.

## Ethics Statement

This survey study aimed to collect different stakeholder opinions, and therefore, did not involve the sharing of sensitive data. Consequently, this study did not require ethical approval. All experts involved in the project were informed of the study’s objectives and the possibility of publishing the results in a peer-reviewed article. Participation was voluntary by invitation and participants did not receive any compensation or benefits for participating in the survey. Participants, by accessing the secure online survey platform using their credentials, gave their consent to participate in the survey by clicking on the appropriate button to submit the completed questionnaire. All survey results are anonymous and presented in aggregate form.

## Author contributions

AV: Conceptualization, Data curation, Methodology, Supervision, Validation, Visualization, Writing – review & editing. SB: Conceptualization, Data curation, Investigation, Methodology, Supervision, Validation, Writing – review & editing. MP: Data curation, Methodology, Supervision, Validation, Writing – review & editing. ES: Data curation, Formal analysis, Writing – original draft. JL: Data curation, Formal analysis, Supervision, Validation, Writing – original draft. LB: Conceptualization, Data curation, Funding acquisition, Investigation, Methodology, Project administration, Supervision, Validation, Writing – review & editing. MP: Conceptualization, Methodology, Supervision, Validation, Writing – review & editing. RI: Conceptualization, Funding acquisition, Investigation, Project administration, Supervision, Writing – review & editing.
